# Transwell migration assay to interrogate human CAR-T cell chemotaxis

**DOI:** 10.1016/j.xpro.2022.101708

**Published:** 2022-09-20

**Authors:** Arman Oner, Sebastian Kobold

**Affiliations:** 1 Division of Clinical Pharmacology, Department of Medicine IV, Klinikum der Universität München, LMU Munich, Munich, Germany; 2 Einheit für Klinische Pharmakologie (EKLiP), Helmholtz Zentrum München, Research Center for Environmental Health (HMGU), Neuherberg, Germany; 3 German Center for Translational Cancer Research (DKTK), Partner Site Munich, Munich, Germany

**Keywords:** Cell isolation, Cell-based assays, Cancer, Immunology, Biotechnology and bioengineering

## Abstract

A major impediment to effective cellular therapies in solid tumors is the limited access of therapeutic cells to the tumor site. One strategy to overcome this challenge is to endow T cells with chemotactic properties required to access tumor tissue. Here, we present a chimeric antigen receptor (CAR)-modified T cell strategy centered around enhanced T cell trafficking. We outline isolation, activation, and transduction of human T cells, as well as techniques for assessing migratory and cytotoxic capacity of CAR-T cells.

For complete details on the use and execution of this protocol, please refer to Lesch et al. (2021).

## Highlights

• Strategy to increase CAR-T cell trafficking into tumor sites
• Assessment of chemotactic potential of CAR-T cells
• Increase in cytotoxicity congruent with enhanced cell migration

## Abstract (editor highlights)

Publisher’s note: Undertaking any experimental protocol requires adherence to local institutional guidelines for laboratory safety and ethics.

## Graphical abstract

**Figure:**
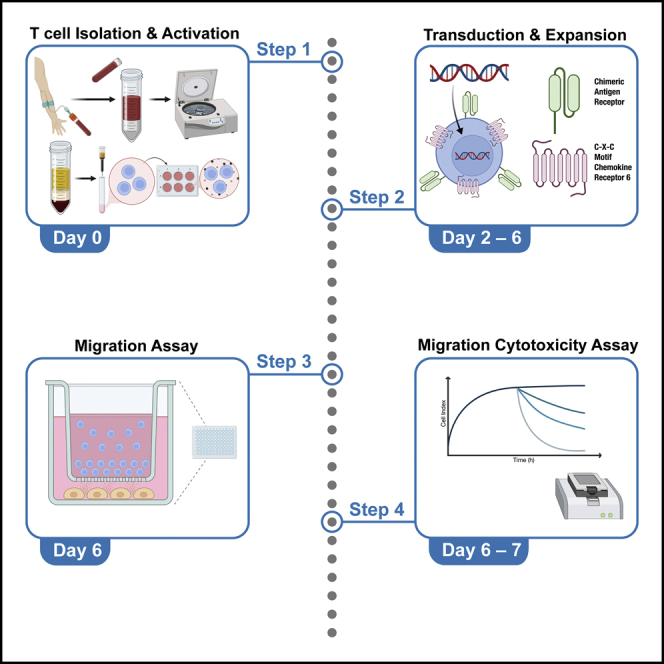

## Before you begin

The protocol below describes the strategy we implemented to increase T cell trafficking into solid tumors. Specifically, we made use of the C-X-C motif chemokine receptor 6, (CXCR6), expressed alongside a chimeric antigen receptor targeting mesothelin, a cell surface glycoprotein, which, among others, has been found to be highly expressed in pancreatic tumors. The assessment tools detailed here, although described in this specific context, can be adapted to determine the migratory potential of otherwise modified T cells (i.e., another chemokine receptor, such as CCR8) or T cells in general (Cadilha et al., 2021). Along the same lines, the chemoattractant detailed here, CXCL16, the exclusive ligand of CXCR6, can be replaced with another chemoattractant of choice.

### Institutional permissions

Processing of whole blood, use of packaging cell lines, and transduction of human T cells must be carried out in a biosafety level 2 (or higher) facility. Work with primary human T cells and BSL-2 retroviruses detailed here was approved by a local ethics committee, as well as a local gene and biological safety agency. Whenever necessary, permissions must be sought from relevant regulatory authorities prior to implementing aspects of this approach or adopting it fully.

### Reconstitute and aliquot reagents, thaw cell lines

**Timing: 1 h**

1. Reconstitute, aliquot and store following reagents according to the manufacturer’s instructions.
  a. Recombinant human interleukin-2, recombinant human interleukin-15, recombinant human CXCL16, Retronectin, 2-mercaptoethanol, l-glutamine and pen/strep.
2. Thaw and expand cell lines.
  a. Thaw one cryovial each of 293Vec-Galv and 293Vec-RD114 by placing them in a water bath warmed to 37°C (Ghani et al., 2009). Take the cryovials out right before they have thawed completely.
  b. Transfer the contents of the cryovials into separate 50 mL conical tubes, each filled with 20 mL of cell culture medium.
  c. Centrifuge the tubes at 400 × g for 5 min.
  d. Resuspend the cell pellets in 10 mL of cell culture medium each and transfer into T-75 cell culture flasks.
  e. Incubate the cells at 37°C with 5% CO_2_. Passage the 293Vec cells whenever necessary by gently tapping on the cell culture flask, removing 90% of the volume and replacing with fresh cell culture medium.

### Generate stable packaging cell lines

**Timing: 5 days + ∼4 weeks (cell culture)**

This section briefly describes the generation of stably transduced packaging cell lines, the viral supernatant of which can be used to engineer T cells to express a desired gene product. The migration assay detailed here compares two sets of anti-MSLN-CAR-T cells, one of which overexpresses CXCR6. Follow the instructions below to generate stably transduced cell lines that code for anti-MSLN-CAR and anti-MSLN-CAR-2A-CXCR6 respectively.

***Note:*** There are a number of commercial actors that offer high-titer virus as service. Also, many institutions have in-house core facilities that can provide investigators with the desired viral products. Make sure the viral plasmid you intend to use is of sufficient quantity and quality (e.g., verification by spectrophotometer and DNA sequencing).

3. (Day 1) Seed 293Vec-Galv cells.
  a. Harvest 293Vec-Galv cells in their exponential growth phase, resuspend the cells at a density of 0.4 × 10^6^ cells/mL in cell culture medium and deposit 2 mL/well into a 6-well plate. Incubate the cells for 16–24 h at 37°C with 5% CO_2_.
4. (Day 2) Transfect 293Vec-Galv cells with the plasmid of interest. In this case with pMP71-anti-MSLN-CAR and pMP71-anti-MSLN-CAR-2A-CXCR6, respectively.
  a. In separate tubes, dilute 10 μL of Lipofectamine 2000 and 4 μg of plasmid DNA in 250 μL of serum-free medium (e.g., Opti-MEM Reduced Serum Medium). Let the diluted lipid particles incubate on the bench (i.e., at 20°C) for 5 min.
  b. Combine the diluted Lipofectamine 2000 with the diluted plasmid DNA, mix well by pipetting up and down several times. Let it incubate on the bench for 20 min.
  c. Add the DNA-lipid complexes dropwise onto the 293Vec-Galv cells, which should have reached 70%–80% confluency. Gently swirl the plate in a figure-of-eight motion. Incubate for 40–48 h at 37°C with 5% CO_2_.

***Note:*** Although it is not necessary to remove the DNA-lipid complexes, this can be done after 4–6 h of incubation.

5. (Day 3) Seed 293Vec-RD114 cells.
  a. Harvest 293Vec-RD114 cells in their exponential growth phase, resuspend the cells at a density of 0.4 × 10^6^ cells/mL in cell culture medium and deposit 2 mL/well into a 6-well plate. Incubate the cells for 16–24 h at 37°C with 5% CO_2_.
6. (Day 4) Transduce previously seeded 293Vec-RD114 cells.
  a. Collect the virus-containing supernatant from 293Vec-Galv cells and pass it through a sterile filter with a pore size of 0.45 μm.
  b. Add an equal amount of fresh cell culture medium to the virus containing supernatant to replenish the medium. Fortify this mixture with 8 μg/mL of polybrene and put it aside.
  c. Bring the 293Vec-RD114 cells, which were seeded the day before, into the laminar flow hood. Carefully remove the medium of 293Vec-RD114 cells. Replenish with the viral supernatant that was set aside.
  d. Incubate the 293Vec-RD114 cells with virus-containing supernatant for 20–24 h at 37°C with 5% CO_2_.

***Note:*** 293Vec-RD114 cells detach easily, so special care must be taken during the medium exchange. This step can also be performed in a reduced serum environment to enhance efficiency.

7. (Day 5) Isolate single clones.
  a. Isolate single clones by serial dilution or Fluorescence Activated Cell Sorting (FACS) into a TC-treated flat bottom 96-well plate.
  b. Incubate the cells for a few weeks at 37°C with 5% CO_2_ until they proliferate enough to warrant a transfer into a bigger well.

***Note:*** Once enough single clones have been generated, they can be functionally validated to identify a suitable clone.

8. (After validation) Harvest and freeze viral supernatant.
  a. Harvest viral supernatant of selected clones. Pass the supernatant through a sterile filter with a pore size of 0.45 μm. Aliquot, flash-freeze, and store at −80°C for later use.

***Note:*** The viral supernatant can be stored for up to a year without significant reduction in transduction capacity. Avoid freeze-thaw cycles when using frozen viral supernatant.

## Key resources table

**Table:** 

REAGENT or RESOURCE	SOURCE	IDENTIFIER
**Antibodies**		

APC anti-human CD8 Antibody (1:100 dilution)	BioLegend	Cat# 344722, RRID: AB_2075388
c-myc Antibody, anti-human/mouse/rat, FITC (1:100 dilution)	Miltenyi Biotec	Cat# 130-116-485, RRID: AB_2751321

**Biological samples**		

Human blood	Healthy Donors	N/A

**Chemicals, peptides, and recombinant proteins**		

2-Mercaptoethanol	Carl Roth	Cat# 4227.3
Aldesleukin (recombinant human IL-2)	Clinigen	Cat# 05060229220264
Bovine Serum Albumin	Carl Roth	Cat# 3854.5
DMEM	Sigma-Aldrich	Cat# D5671-500ML
DPBS	Sigma-Aldrich	Cat# D8537-500ML
EDTA	Sigma-Aldrich	Cat# 03690-100ML
Fetal Bovine Serum	Gibco	Cat# 16000044
Histopaque-1077	Sigma-Aldrich	Cat# 10771-500ML
Human Serum	Sigma-Aldrich	Cat# H4522-100ML
L-Glutamine	Sigma-Aldrich	Cat# G7513-100ML
Lipofectamine 2000	Thermo Fisher Scientific	Cat# 11668019
MEM Non-Essential Amino Acids Solution (100×)	Gibco	Cat# 11140050
Opti-MEM I Reduced Serum Medium	Gibco	Cat# 31985070
Penicillin-streptomycin	Sigma-Aldrich	Cat# P4333-100ML
Polybrene	Sigma-Aldrich	Cat# TR-1003-G
Recombinant human CXCL16	PeproTech	Cat# 300-55
Recombinant human IL-15	PeproTech	Cat# 200-15
Retronectin	Takara Bio Inc.	Cat# T100B
RPMI 1640	Sigma-Aldrich	Cat# R0883-500ML
Sodium Pyruvate	Sigma-Aldrich	Cat# S8636-100ML
Trypsin-EDTA Solution 10×	Sigma-Aldrich	Cat# 59418C-100ML

**Experimental models: Cell lines**		

Human: 293Vec-Galv	Manuel Caruso, Quebec, Canada	N/A
Human: 293Vec-RD114	Manuel Caruso, Quebec, Canada	N/A
Human: 293Vec-RD114-anti-MSLN-CAR	Lesch et al. (2021)	N/A
Human: 293Vec-RD114-anti-MSLN-CAR-CXCR6	Lesch et al. (2021)	N/A
Human: SUIT-2-MSLN-CXCL16	Lesch et al. (2021)	N/A

**Recombinant DNA**		

Plasmid: pMP71	C. Baum	N/A
Plasmid: pMP71-anti-MSLN-CAR	Lesch et al. (2021)	N/A
Plasmid: pMP71-anti-MSLN-CAR-CXCR6	Lesch et al. (2021)	N/A

**Software and algorithms**		

Adobe Creative Suite CS6	Adobe	https://www.adobe.com/creativecloud
Biorender	BioRender	https://biorender.com
Flowjo 10	BD	https://www.flowjo.com
GraphPad Prism 9	GraphPad	https://www.graphpad.com
RTCA Software	Agilent	Cat# 5454433001

**Other**		

BD LSRFortessa Cell Analyzer	BD Biosciences	N/A
CD3 MicroBeads, human	Miltenyi Biotec	Cat# 130-050-101
CountBright™ Absolute Counting Beads	Thermo Fisher Scientific	Cat# C36950
Dynabeads Human T-Activator CD3/CD28	Gibco	Cat# 11132D
E-Plate 96	Agilent	Cat# 5232376001
HTS Transwell-96 Permeable Support with 3.0 μm Pore Polycarbonate Membrane	Corning	Cat# 3386
HTS Transwell-96 Receiver Plate, Clear, Sterile	Corning	Cat# 3382
LS Column Adapter	Miltenyi Biotec	Cat# 130-090-544
MACS MultiStand	Miltenyi Biotec	Cat# 130-042-303
QuadroMACS Separator	Miltenyi Biotec	Cat# 130-090-976
xCELLigence RTCA SP System	Agilent	Cat# 00380601030

## Materials and equipment

**Table:** Cell culture medium

Reagent	Final concentration	Amount
FBS	10%	50 mL
L-glutamine	4 mM	10 mL
Penicillin-streptomycin	100 U/mL and 100 μg/mL, respectively	5 mL
DMEM	N/A	435 mL
**Total**	**N/A**	**500 mL**

Store at 4°C for 4–6 weeks.

**Table:** Cytotoxicity medium

Reagent	Final concentration	Amount
FBS	10%	50 mL
L-glutamine	2 mM	5 mL
Penicillin-streptomycin	100 U/mL and 100 μg/mL, respectively	5 mL
MEM non-essential amino acids (100×)	1×	5 mL
Sodium pyruvate solution 100 mM	1 mM	5 mL
DMEM	N/A	430 mL
**Total**	**N/A**	**500 mL**

Store at 4°C for 4–6 weeks.

**Table:** Human T cell medium

Reagent	Final concentration	Amount
Human serum	2.5%	12.5 mL
L-glutamine	2 mM	5 mL
Penicillin-streptomycin	100 U/mL and 100 μg/mL, respectively	5 mL
MEM non-essential amino acids (100×)	1×	5 mL
Sodium pyruvate solution 100 mM	1 mM	5 mL
RPMI 1640 medium	N/A	467.5 mL
**Total**	**N/A**	**500 mL**

Store at 4°C for 4–6 weeks.

**Table:** Migration medium

Reagent	Final concentration	Amount
Human Serum	1%	5 mL
L-glutamine	2 mM	5 mL
Penicillin-streptomycin	100 U/mL and 100 μg/mL, respectively	5 mL
MEM non-essential amino acids (100×)	1×	5 mL
Sodium pyruvate solution 100 mM	1 mM	5 mL
RPMI 1640 medium	N/A	475 mL
**Total**	**N/A**	**500 mL**

Store at 4°C for 4–6 weeks.

**Table:** Tumor cell line medium

Reagent	Final concentration	Amount
FBS	10%	50 mL
L-glutamine	2 mM	5 mL
Penicillin-streptomycin	100 U/mL and 100 μg/mL, respectively	5 mL
DMEM	N/A	440 mL
**Total**	**N/A**	**500 mL**

Store at 4°C for 4–6 weeks.

**Table:** Blocking buffer

Reagent	Final concentration	Amount
BSA	2%	1 g
PBS, pH 7.4	N/A	50 mL
**Total**	**N/A**	**50 mL**

Store at 4°C for 4–6 weeks.

**Table:** MACS buffer

Reagent	Final concentration	Amount
BSA	0.5%	2.5 g
EDTA, 0.5 M	2 mM	2 mL
PBS, pH 7.4	N/A	498 mL
**Total**	**N/A**	**500 mL**

Store at 4°C for 4–6 weeks.

**Table:** FACS buffer

Reagent	Final concentration	Amount
BSA	0.5%	2.5 g
EDTA, 0.5 M	2 mM	2 mL
PBS, pH 7.4	N/A	498 mL
**Total**	**N/A**	**500 mL**

Store at 4°C for 4–6 weeks.

## Step-by-step method details

### Isolation of human peripheral blood T cells

**Timing: 3 h**

This section briefly describes the isolation of peripheral blood mononuclear cells (PBMCs) from whole blood and pan T cell enrichment that follows. When isolating PBMCs a variety of different starting materials may be used (e.g., whole blood, buffy coat, leukoreduction system chamber, etc.). The procedure described here may need to be adjusted depending on the cell density of these alternative products.

**CRITICAL:** Follow national regulations as well as institutional guidelines regarding biohazardous materials when handling whole blood.

***Note:*** Unless otherwise specified, all steps are to be carried out in a laminar flow hood, using proper aseptic technique.

**Figure 1:**
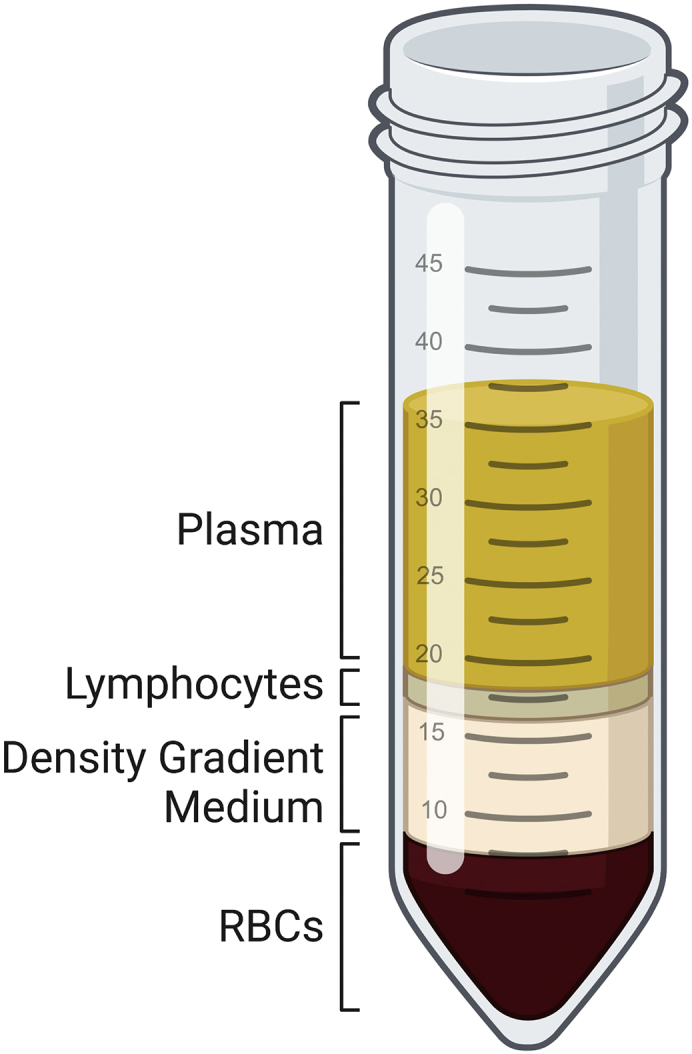
Whole blood after density gradient centrifugation Upon centrifugation there should be four distinct layers in each tube. From top to bottom: plasma, PBMCs, density gradient medium and red blood cells.

1. Determine the volume of whole blood to be processed.
  a. Calculate the number of T cells to be transduced on day 2, add one to account for a non-transduced condition and multiply by 1.8.
2. Isolate PBMCs using density gradient centrifugation.
  a. Dilute the whole blood by adding one part PBS. Mix gently with a serological pipette.
  b. Dispense 15 mL of density gradient medium (such as Histopaque-1077) into sterile 50 mL conical tubes (each tube can process 35 mL of diluted blood).
  c. Carefully layer the diluted blood onto the density gradient medium. This is best achieved by holding the tube at a 45° angle, leaning against the side of the tube with the pipette tip and slowly releasing the diluted blood.
    ***Note:*** Take care not to disrupt the layer between the density gradient medium and the diluted blood. Over time some red blood cells may have already settled to the bottom of the tube prior to centrifugation. This is expected, proceed with the centrifugation as described.
  d. Centrifuge the samples at 1,000 × *g* for 20 min at 20°C in a swinging-bucket rotor. Make sure the brake is set to 0 (Figure 1).
  e. Using a serological pipette carefully transfer each PBMC layer into new 50 mL conical tubes. Fill the tubes with PBS before centrifuging at 520 × g for 15 min at 20°C (default brake settings may be reapplied).
  f. Aspirate the supernatant and discard. Resuspend the cell pellets in 5 mL of PBS each and pool PBMCs from the same donor into a single 50 mL conical tube. Fill the tube with PBS and note the cell count of PBMCs using a hemocytometer or an automated cell counter.
3. Isolate pan T cells by magnetic enrichment.
  a. Centrifuge the tube at 520 × g for 10 min at 20°C.
  b. Aspirate the supernatant and discard. Resuspend the cell pellet in 80 μL of cold MACS buffer per 10^7^ cells.
  c. Resuspend CD3 Microbeads thoroughly by vortexing for 15 s and add 20 μL of beads per 10^7^ cells. Mix the sample well by pipetting up and down. Incubate for 15 min at 4°C.
  d. Subsequently add 2 mL of cold MACS buffer per 10^7^ cells and centrifuge the sample at 300 × g for 10 min at 4°C.
  e. In the meantime, prepare for cell separation by attaching an LS Column onto a compatible MACS separator. Put a 50 mL conical tube under the column to catch the flow through and apply 3 mL of cold MACS buffer onto the column.
  f. Once the centrifuge is done, aspirate the supernatant and discard. Resuspend the cell pellet in 500 μL of cold MACS buffer. Add another 50 μL of buffer for every 10^7^ cells exceeding the first 10^8^ cells.
  g. Load the cell suspension onto the prepared LS Column.
  h. After it flows through completely wash the column with 3 mL of cold MACS buffer. Repeat this step two more times.
  i. Discard the flow through and put a new 50 mL conical tube under the column.
  j. Apply 5 mL of cold MACS buffer and immediately afterwards put on the plunger. Maintain a moderate continuous push to recover the labeled pan T cells.
  k. Add 25 mL of cold MACS buffer and perform a cell count.

### Activation of pan T cells

**Timing: 2 days**

This step enables the efficient transduction of T cells, as γ-retroviruses are able to transduce dividing cells only.

4. Prepare pan T cells for transduction by providing them with appropriate stimuli.
  a. Determine the amount of medium required to achieve a cell density of 10^6^ cells/mL and prepare enough c-hTcm (complete human T cell medium) by supplementing hTcm (human T cell medium) with 200 U/mL (14.45 ng/mL) IL-2, 5 ng/mL IL-15 and 50 μM 2-ME.
    ***Note:*** c-hTcm should be prepared freshly each time.
  b. Resuspend CD3/CD28 T cell Activator Dynabeads by vigorously vortexing the vial for 30 s.
  c. Aiming for a bead-to-cell ratio of 1:3, transfer the volume required into a tube.
  d. Wash by adding an equal amount or 1 mL of PBS, whichever is higher. Put the beads in PBS on a magnet for 1 min and remove the liquid, leaving the beads behind.
  e. Resuspend the beads in the same volume of c-hTcm as initially transferred.
    ***Note:*** Alternative activation methods (i.e., stimulatory beads from other vendors or antibody coated plates, etc.) are also viable.
  f. Centrifuge pan T cells at 400 × g for 10 min at 20°C. Aspirate the supernatant and discard.
  g. Resuspend the cell pellet in c-hTcm at a concentration of 10^6^ cells/mL.
  h. Seed pan T cells in a 6-well plate in a final volume of 3–4 mL/well.
  i. Dispense the prepared beads into individual wells at a final bead-to-cell ratio of 1:3. Make sure that beads are well-dispersed by pipetting up and down.
  j. Incubate the cells for 48 h at 37°C with 5% CO_2_.

### Transduction and expansion of pan T cells

**Timing: 2–4 days**

**Figure 2:**
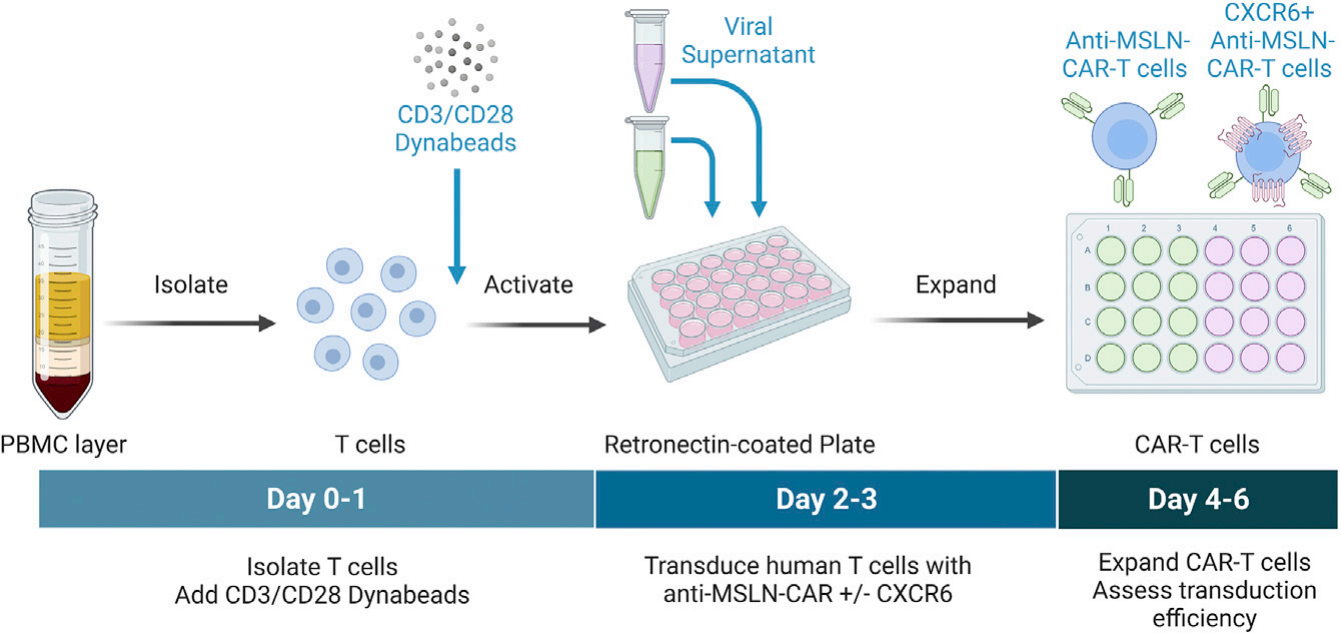
Graphical depiction of the transduction workflow

This section deals with T cell transduction using a γ-retroviral delivery system as an example. Other gene editing techniques, viral or otherwise, might be utilized to achieve similar results. At the end of this section, T cells should stably express the anti-MSLN-CAR along with CXCR6 on their surface. Control cells that only express the anti-MSLN-CAR and non-transduced cells should also be generated (Figure 2).

5. (A day before transduction) Prepare Retronectin -coated plates.
  a. Prepare a 6.25 μg/mL solution of Retronectin diluted in PBS.
  b. Dispense 400 μL of diluted Retronectin into wells of a non-tissue treated 24-well plate. Incubate at 4°C for 16–20 h.
6. (On the day of transduction) Prepare virus and the Retronectin -coated plates for transduction.
  a. Aspirate and discard Retronectin solution in the 24-well plate prepared the day before. Promptly add 500 μL of blocking buffer (2% BSA in PBS) into each Retronectin-coated well. Incubate on the bench for 30 min.
  b. In the meantime, thaw viral supernatant of constructs that will be used.
  c. After 30 min of blocking, remove the blocking buffer and wash the wells with 1 mL of PBS before depositing 1 mL of viral supernatant to wells that will be used for transduction.
  d. Centrifuge the loaded plate at 3,000 × *g* for 1.5 h at 32°C.

***Note:*** Check the limitations of the specific centrifuge intended for use beforehand to ensure a safe workplace.

7. Transduce and expand T cells.
  a. Examine the isolated T cells under an inverted tissue-culture microscope. T cells should be blasting and forming clusters.
  b. Resuspend the stimulated T cells well by pipetting up and down. Transfer them into a 50 mL conical tube and perform a cell count.
  c. Centrifuge the cells at 400 × g for 5 min at 20°C. Aspirate the supernatant and discard.
  d. Resuspend the cell pellet in freshly supplemented c-hTcm at a concentration of 10^6^ cells/mL.
  e. After 1.5 h of centrifugation remove the viral supernatant from wells of the now virus-coated Retronectin plate and promptly deposit 1 mL of T cells suspended in fresh c-hTcm.
  f. Return to a 37°C incubator. Every other day perform a cell count and subculture cells in fresh c-hTcm. Check transduction efficiency as early as 72 h after transduction using a flow cytometer.

***Note:*** Transduction efficiency can be determined using a variety of methods (e.g., a bicistronic vector that codes for a fluorescent protein along with the gene of interest, an epitope tag, etc.). The CAR constructs described in this work incorporate a MYC tag, which enables the identification of transduced T cells by fluorescently labeled antibodies.

### Transwell migration assay

**Timing: 8 h**

The transwell migration assay evaluates cell migration through the use of a chemotactic gradient. In this form of assay, a cell-permeable membrane is placed between an upper and a lower chamber. Cells of interest are then seeded in the upper chamber, whereas the chemoattractant in question is deposited into the lower chamber. At the end of the assay, cells that have migrated specifically are quantified to derive a chemotactic index.

***Note:*** The number of cells to be disposed into the upper chamber as well as the concentration of the chemoattractant and incubation time may need to be adjusted based on the question that motivates the experiment.

8. Prepare required assay media (incl. positive and baseline controls).
  a. Dilute the chemoattractant of interest, in this case, recombinant human CXCL16 in migration medium to a final concentration of 50 ng/mL.
  b. As a positive control, prepare migration medium with 10% human serum. Use migration medium alone to establish a baseline. Volume required = 225 μL/well.

***Note:*** The relationship between the concentration of a particular chemoattractant and its ability to induce migration is not sigmoidal but rather bell-shaped. Therefore, it is crucial to determine the suitable concentration range for the chemoattractant in question prior to this migration assay. The range can be estimated reasonably by reviewing the literature along with the information provided by the vendor of the chemoattractant in question. Test out an order of magnitude above and below as a means of optimization.

9. Prepare T cells for the assay.
  a. Harvest T cells and transfer them into 50 mL conical tubes, pooling cells from the same conditions.
  b. Perform a cell count of each condition.
  c. Centrifuge the cells at 400 × g for 5 min at 20°C. Aspirate the supernatant and discard.
  d. To minimize serum carryover, resuspend the cell pellet in 30 mL of PBS and centrifuge at 400 × g for 5 min at 20°C. Aspirate the supernatant and discard. Repeat this step one more time.
  e. Resuspend the cells in migration medium at a concentration of 1 × 10^6^ cells/70 μL (∼14.29 × 10^6^ cells/mL).
10. Set up the migration assay.
  a. Take a transwell migration plate into a laminar flow hood.
  b. Remove the lid and deposit 70 μL of cell suspension into the upper chamber (also called the insert) of the transwell plate.
  c. Without removing the insert, add 225 μL of assay medium into the lower chamber.
    ***Note:*** Employ a reverse pipetting technique to prevent bubble formation. Push the knob all the way down to the second stop, aspirate the sample and dispose by pushing the knob until the first stop. Repeat as often as needed.
    **CRITICAL:** Bubbles trapped under the insert disrupt cell migration and may lead to inconsistent results.
  d. Put the lid on gently without applying any pressure and transfer the plate to an incubator. Let the cells migrate for 4 h.
    ***Note:*** While moving the assay plate make sure not to make any sudden or jerky movements. Likewise, minimize any disruptions that may take place during the assay (e.g., moving the plate around, agitation of the plate by shutting the incubator forcefully, temperature swings caused by excessive use of the incubator, etc.).
11. Read-out by FACS.
  a. Take out the transwell plate carefully and remove the lid.
  b. Use cotton swabs to get rid of any remaining liquid in the upper chamber to minimize the risk of spillover. Take care not to damage the membrane.
  c. Disassemble the transwell plate carefully by holding the plate firmly in place with one hand, while removing the insert with the other.
  d. Lift the cells by pipetting up and down and transfer the cells into a round bottom 96-well plate. Centrifuge at 400 × g for 5 min at 20°C.
  e. Discard supernatant. Resuspend the cells in 200 μL of FACS buffer. Centrifuge at 400 × g for 5 min at 20°C. Repeat once more.
  f. Prepare a master mix of fluorescently labeled antibodies in FACS buffer to stain receptors of interest.
  g. Discard supernatant and resuspend the cells in the prepared master mix. Incubate at 4°C for 20 min.
  h. Centrifuge at 400 × g for 5 min.
  i. Discard supernatant. Resuspend the cells in 200 μL of FACS buffer. Centrifuge at 400 × g for 5 min at 20°C. Repeat once more.
  j. Discard supernatant and resuspend the cells in 100 μL of FACS buffer.
  k. Samples are ready for acquisition.

***Optional:*** Prior to acquisition, counting beads may be added to the cell suspension to quantify the migrated cells. Ensure accurate cell counts by following the instructions for the specific product you plan to use. Other quantifications methods, such as the use of a volumetric flow cytometer, are also valid.

### Migration cytotoxicity assay

**Timing: 2–4 days**

**Figure 3:**
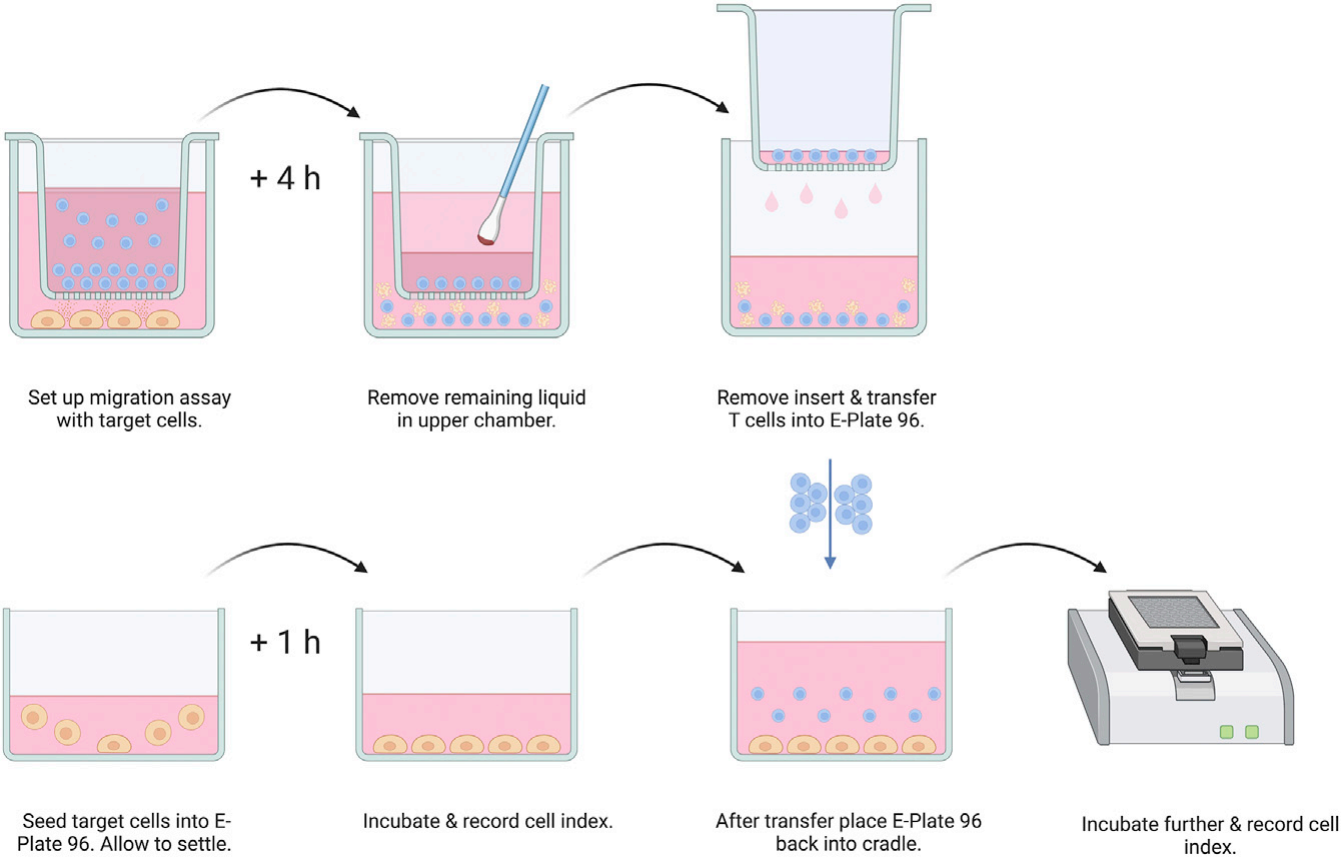
Illustration of a combined migration cytotoxicity assay The upper part of the illustration depicts a migration assay *(ref. steps 8–11)*, whereas the lower part outlines an impedance-based cytotoxicity assay *(ref. steps 12–15)*.

This section combines the transwell migration assay with an impedance-based real-time cell analysis (RTCA) tool, which uses impedance as a proxy for the number of target cells and their overall health (Figure 3). Impedance readings are processed to derive a unitless cell index that can be plotted against time to visualize cell-mediated cytotoxicity. Using this approach, investigators can introduce a new dimension to their analysis by examining the cytotoxic capacity of CAR-T cells in addition to their migratory potential. The target cell line used in this section is an engineered SUIT-2 clone that overexpresses MSLN and CXCL16. SUIT-2-MSLN-CXCL16 cells are to be cultured in tumor cell line medium and maintained well before use.

***Note:*** The impedance-based assay described in this section permits the use of adherent as well as non-adherent cell lines. The use of non-adherent cell lines, however, requires additional steps not described in this section.

12. Establish baseline and seed target cells.
  ***Note:*** The success of this impedance-based assay depends heavily on the quality of target cells in question. It is crucial that target cells be in logarithmic growth phase and have a low passage number.
  a. Prewarm reagents required for this step to 37°C (i.e., cytotoxicity medium and 1× trypsin-EDTA).
  b. Take an E-Plate 96 into a laminar flow hood and deposit 50 μL of cytotoxicity medium or PBS into all wells.
    ***Note:*** It is recommended to use up the entire E-Plate 96 for an experiment. Wells that are not needed can be filled with PBS instead.
  c. Place the E-Plate into the RTCA cradle.
  d. Using the control unit (a laptop) perform background measurement (step 1) on the E-Plate 96. Subsequently, take out the E-Plate 96 and bring it back into the laminar flow hood for cell seeding.
  e. Prepare target cells (e.g., SUIT-2-MSLN-CXCL16 cells) by removing their medium, washing them with PBS and treating them with 1× trypsin-EDTA. Incubate at 37°C, checking on the cells every minute. Add serum-containing medium as soon as 90% of the cells have detached.
    ***Note:*** Trypsin exposure of cells should be kept to a minimum and must be halted promptly with serum-containing medium upon detachment of cells. Prolonged periods of trypsin exposure may compromise the performance of the assay.
  f. Transfer the cells into a 50 mL conical tube and centrifuge at 400 × g for 5 min at 20°C. Aspirate the supernatant and discard. Resuspend the cell pellet in cytotoxicity medium.
    ***Note:*** The optimal number of cells per well is dictated by the target cell line in question and other site-specific factors. For most cell lines this number should fall between 5,000 and 100,000 cells per well and must be determined empirically.
  g. Using a multichannel pipette and reagent reservoir, add 100 μL of cell suspension into the wells of the E-Plate 96. Resuspend regularly to prevent uneven cell seeding.
  h. Let the target cells settle by leaving the E-Plate 96 on the bench for 45–60 min.
  i. Return the E-Plate 96 to the RTCA cradle. Using the control unit initiate step 2 of the RTCA experiment. Incubate until an appropriate Cell Index has been reached (e.g., 1–2).
    **CRITICAL:** Be generous with the duration of RTCA steps, as shorter than necessary steps can lead to a pause causing a gap in the cell index curve. Longer than necessary steps can be skipped without any disruption.
13. Run a transwell migration assay.
  ***Note:*** Following steps have been modified to allow for the use target cells. Be aware that this section requires the use of a tissue culture treated transwell receiver plate, which needs to be obtained separately.
  a. Seed 2.5 × 10^4^ SUIT-2-MSLN-CXCL16 cells/well in a final volume of 225 μL of cytotoxicity medium in a TC-treated transwell receiver plate. Incubate for 24 h at 37°C with 5% CO_2_.
    ***Note:*** Time the following migration assay based on the slope of the CI curve, so that by the end of the assay the Cell Index reaches a value between 1 and 2.
  b. After 24 h of incubation time, follow previously described instructions with changes to steps 9.e and 10.a–c to run a migration assay (repeated here for readability).
    i. Harvest T cells and transfer them into 50 mL conical tubes, pooling cells from the same conditions.
    ii. Perform a cell count of each condition.
    iii. Centrifuge the cells at 400 × g for 5 min at 20°C. Aspirate the supernatant and discard.
    iv. Resuspend the cell pellet in 30 mL of PBS and centrifuge at 400 × g for 5 min at 20°C. Aspirate the supernatant and discard.
    v. Resuspend the cells in cytotoxicity medium at a concentration of 5 × 10^5^ cells/70 μL (∼7.14 × 10^6^ cells/mL).
    vi. Take the TC-treated receiver plate with chemoattractant-secreting cells and a transwell migration plate into a laminar flow hood.
    vii. Remove the lids of both plates. Carefully move the transwell insert and place it onto the TC-treated receiver plate. Deposit 70 μL of cell suspension into the upper chamber.
    viii. Put the lid on gently without applying any pressure and transfer the plate to an incubator. Let the cells migrate for 4 h.
14. Seed effector cells.
  a. Confirm that the Cell Index is in the desired range.
  b. Take out the transwell plate carefully and remove the lid.
  c. Use cotton swabs to get rid of any remaining liquid in the upper chamber to minimize the risk of spillover. Take care not to damage the membrane.
  d. Disassemble the transwell plate carefully by holding the plate firmly in place with one hand, while removing the insert with the other.
  e. Use the control unit to abort the Step currently running (step 2) and start the next one. After the first sweep pause the experiment immediately.
  f. Carefully release and remove the E-Plate 96 from the cradle. Subsequently, bring the E-Plate 96 into the laminar flow hood and remove its lid.
  g. Resuspend the cells in the lower chamber of the transwell plate by pipetting up and down and transfer 100 μL of the migrated cells into the wells of the E-Plate 96.
  h. Put the lid back on and return the E-Plate 96 into the cradle.
  i. Press continue on the control unit to continue the experiment.
15. FACS the remaining cells of the completed migration assay.
  a. Use 100 μL of the remaining cell suspension in the lower chamber and follow steps 11.d to 11.k (repeated here for readability).
    i. Lift the cells by pipetting up and down and transfer the cells into a round bottom 96-well plate. Centrifuge at 400 × g for 5 min at 20°C.
    ii. Discard supernatant. Resuspend the cells in 200 μL of FACS buffer. Centrifuge at 400 × g for 5 min at 20°C. Repeat once more.
    iii. Prepare a master mix of fluorescently labeled antibodies in FACS buffer to stain receptors of interest.
    iv. Discard supernatant and resuspend the cells in the prepared master mix. Incubate at 4°C for 20 min.
    v. Centrifuge at 400 × g for 5 min.
    vi. Discard supernatant. Resuspend the cells in 200 μL of FACS buffer. Centrifuge at 400 × g for 5 min at 20°C. Repeat once more.
    vii. Discard supernatant and resuspend the cells in 100 μL of FACS buffer.
    viii. Samples are ready for acquisition.

## Expected outcomes

The approach described here, when successfully implemented, is expected to result in increased trafficking of T cells into tumor sites, which is expected to lead to increased anti-tumor activity and enhanced tumor control (Stoiber et al., 2019, Figure 4).

**Figure 4:**
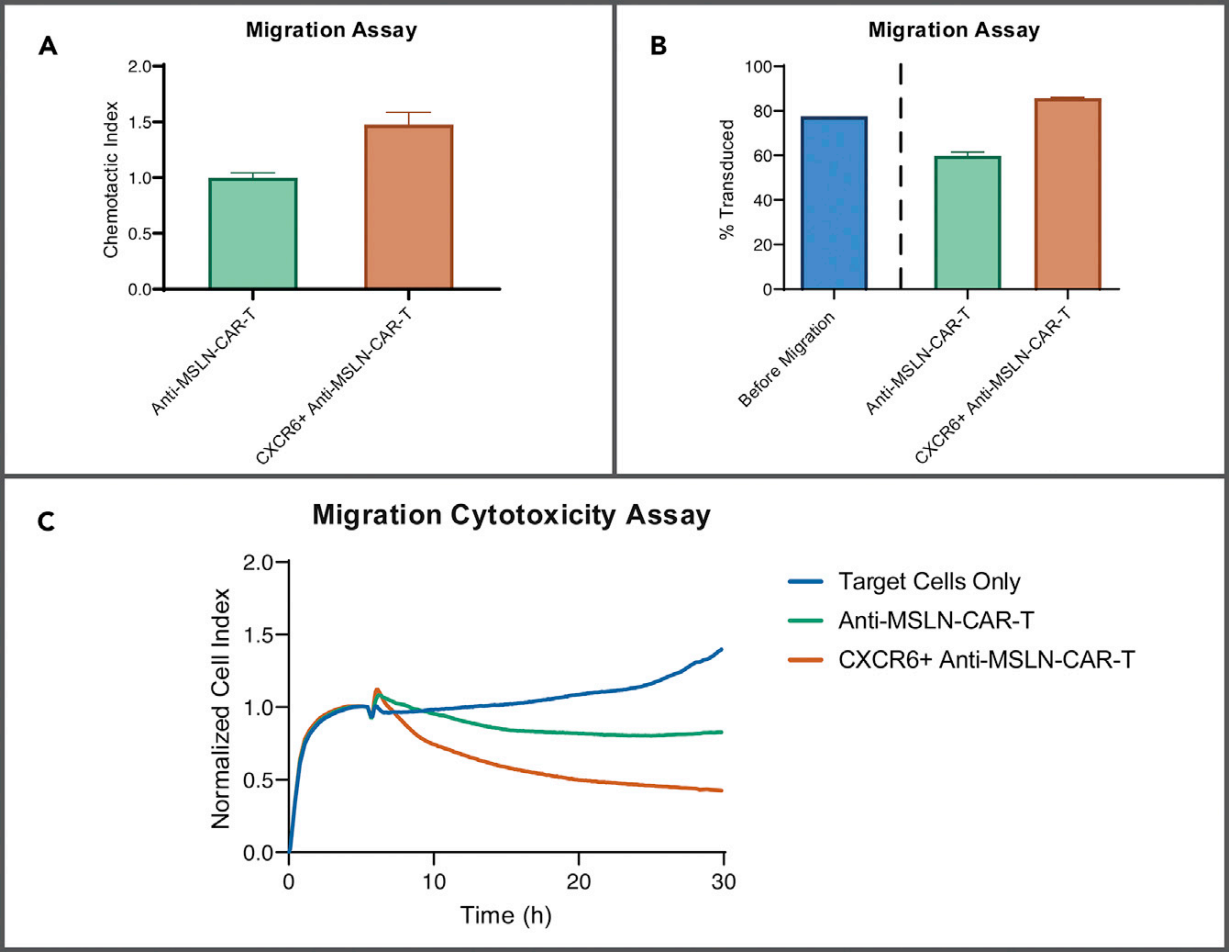
Expected outcomes Results depicting an exemplary migration cytotoxicity assay. (A) Comparison of migratory potential of Anti-MSLN-CAR-T cells and CXCR6+ Anti-MSLN-CAR-T cells towards SUIT-2-MSLN-CXCL16 cells (mean ± SEM). Chemotactic Index was derived using Anti-MSLN-CAR-T cells as baseline. (B) The percentage of transduced cells is depicted. Blue bar represents the percentage of transduced cells deposited into the upper chamber at the start of the assay for both conditions. Green and orange bars represent CAR only and CXCR6+ CAR conditions at the end of the assay, respectively (mean ± SEM). (C) Following the migration assay, a fraction of the migrated cells is used to perform an impedance-based cytotoxicity assay.

## Quantification and statistical analysis

The Chemotactic Index can be derived by dividing the number of cells in the lower chamber of each condition by the average number of cells in the lower chamber of control conditions.

$$ChI=\frac{x}{\left(\frac{1}{n}\sum_{i=1}^{n}y_{i}\right)}$$

*ChI*: *Chemotactic Index*

*x*: *Number of cells in experimental wells*

*y*: *Number of cells in baseline wells*

Normalized cell index can be calculated by dividing the cell index measurements by the last cell index reading before the cytotoxic treatment (e.g., the addition of CAR-T cells). Each well is considered in isolation.

$$NCI_{w}\left(t\right)=\frac{CI_{w}\left(t\right)}{CI_{w}\left(t_{norm}\right)}$$

*NCI*_*w*_(*t*): *Normalized Cell index of well w at time point t*

*CI*_*w*_(*t*):*Cell index of well w at time point t*

*CI*_*w*_(*t*_*norm*_):*Cell index of well w at time point t*_*norm*_

## Limitations

The method described here does not recapitulate biological complexities in their entirety. Notably, the described methodology is inadequate to assess the influence of the endothelium, cancer associated fibroblasts, and the often-remodeled extracellular matrix on T cell trafficking.

## Troubleshooting

### Problem 1

Low transduction efficiency of T cells *(steps 5–7)*.

### Potential solution

Consider using a previously tested batch of γ-retrovirus that codes for a convenient marker, such as GFP, as positive control. If the selected positive control yields satisfactory results, efforts should be focused on the γ-retrovirus and packaging cell lines. Viral titers can be increased by extending the incubation period of packaging cells prior to harvest and/or by omitting the initial freeze-thaw cycle. A higher titer can also be achieved through ultracentrifugation of the viral supernatant. The transduction efficiency of T cells can be increased by extending the centrifugation period of the Retronectin-coated plate with the viral supernatant. Finally, generation of new packaging cell lines or identification of more efficient single-cell clones are strongly encouraged.

If the selected positive control yields poor results, efforts should be focused on T cells and specific transduction reagents used. It is crucial that T cells be well-stimulated and forming clusters on the day of transduction. Make sure that these conditions are met by inspecting T cells under an inverted tissue-culture microscope. Also, make sure that the specific batch of Retronectin has not been subjected to freeze-thaw cycles and was diluted appropriately.

### Problem 2

Low viability or insufficient expansion of CAR-T cells *(step 4)*.

### Potential solution

A number of factors, such as overstimulation, neglect, or excessive viral titers can lead to low CAR-T cell viability or expansion. Start by ensuring that the number of Dynabeads used does not exceed the recommended bead-to-cell ratio of 1:1. Dynabeads can be titrated to prevent cell death related to overstimulation. Experiment with different bead-to-cell ratios ranging from 1:1 to 1:10. Another measure that can be taken, is to provide T cells with an adequate cytokine support as an anti-apoptotic signal (e.g., IL-15). T cell medium should be supplemented with cytokines freshly each time shortly before use. Finally, excessive viral titers may lead to cytotoxicity. In such cases, dilution of viral titers is recommended.

### Problem 3

Scant T cell migration *(step 10)*.

### Potential solution

Inconsistent or insufficient T cell migration may be due to executional or cell-intrinsic factors. T cells can be sensitized to chemotactic cues by subjecting them to serum-starvation prior to migration. A phenotypic profiling may also prove helpful to ensure that T cells do express the chemokine receptor in question that would enable them to mount a migratory response. If the chemoattractant in question is a recombinant protein, the optimal concentration range must be determined by serial titration. On the executional side, assay duration as well as seeding cell density may be increased to achieve sufficient migration. Be mindful that these adjustments may lead to higher levels of unspecific migration. In addition, while depositing medium into the lower chamber, bubbles must be avoided at all costs.

### Problem 4

Cell Index curve does not reach the desired level / shows a wavy pattern or an unexpected drop *(step 12)*.

### Potential solution

Cell health is a crucial aspect of this impedance-based assay. Ensure that the selected target cell line has been maintained well and is free of contamination. Allow target cells ample time to settle before taking the E-Plate 96 into the incubator. Minimize the use of the incubator during the assay, extended periods of unrestricted access are specifically to be avoided, as they can lead to substantial temperature deviation. Due to the nature of their increased exposure, there can be some variance between the outer wells of the E-Plate 96 and the inner wells. This phenomenon, termed edge effect, can be observed in any microplate, and may be the reason for the observed well-to-well inconsistency. If the variance is too great, fill the outer wells with PBS and confine the assay to the inner wells instead.

### Problem 5

Cell Index of wells that did not receive any cytotoxic compound (i.e., CAR-T cells) shows a drop in signal *(step 12)*.

### Potential solution

This is primarily a sign that the medium has become depleted of nutrients and can no longer support the target cells. For long experiments, the medium should be replenished regularly. Contamination or incubator failure can also be pursued as potential causes.

## Resource availability

### Lead contact

Further information and requests for resources and reagents should be directed to and will be fulfilled by the lead contact, Sebastian Kobold (sebastian.kobold@med.uni-muenchen.de).

### Materials availability

Plasmids and cell lines described in this study will be made available on request, but we may require a payment and/or a completed materials transfer agreement if there is potential for commercial application. Requests for the packaging cell lines 293Vec-Galv and 293Vec-RD114 should be addressed to Manuel Caruso, PhD, Université de Québec, Canada.

## Data Availability

This study did not generate any new datasets or code. Original/source data for figures in the paper are available (Lesch et al., 2021).
